# Anti-HIV Activity of *Ocimum labiatum* Extract and Isolated Pheophytin-a

**DOI:** 10.3390/molecules22111763

**Published:** 2017-11-06

**Authors:** Petrina Kapewangolo, Martha Kandawa-Schulz, Debra Meyer

**Affiliations:** 1 Department of Biochemistry, Faculty of Natural and Agricultural Sciences, University of Pretoria, Pretoria 0002, South Africa; pkapewangolo@unam.na; 2 Department of Chemistry and Biochemistry, Faculty of Science, University of Namibia, P/Bag 13301, Windhoek 9000, Namibia; kschulz@unam.na; 3 Department of Biochemistry, Faculty of Science, University of Johannesburg, Auckland Park, Johannesburg 2006, South Africa

**Keywords:** *Ocimum labiatum*, pheophytin-a, HIV-1 protease, HIV-1 reverse transcriptase, HIV-1 replication, real-time cell electronic sensing

## Abstract

*Ocimum* plants are traditionally used to manage HIV/AIDS in various African countries. The effects of *Ocimum labiatum* extract on HIV-1 protease (PR) and reverse transcriptase (RT) is presented here along with characterization of an identified bioactive compound, achieved through ^1^H- and ^13^C-NMR. The extract’s effect on HIV-1 replication was assessed by HIV-1 p24 antigen capture. Cytotoxicity of samples was evaluated using tetrazolium dyes and real-time cell electronic sensing (RT-CES). *Ocimum labiatum* inhibited HIV-1 PR with an IC_50_ value of 49.8 ± 0.4 μg/mL and presented weak inhibition (21%) against HIV-1 RT. The extract also reduced HIV-1 replication in U1 cells at a non-cytotoxic concentration (25 μg/mL). The CC_50_ value of the extract in U1 cells was 42.0 ± 0.13 μg/mL. The HIV-1 PR inhibiting fraction was purified using prep-HPLC and yielded a chlorophyll derivative, pheophytin-a (phy-a). Phy-a inhibited HIV-1 PR with an IC_50_ value of 44.4 ± 1.5 μg/mL (51 ± 1.7 μM). The low cytotoxicity of phy-a in TZM-bl cells was detected by RT-CES and the CC_50_ value in U1 cells was 51.3 ± 1.0 μg/mL (58.9 ± 1.2 μM). This study provides the first in vitro evidence of anti-HIV activity of *O. labiatum* and isolated phy-a, supporting further investigation of *O. labiatum* for lead compounds against HIV-1.

## 1. Introduction

Natural products are still being explored as potential antiviral agents and more importantly as inhibitors of the various steps of the HIV life cycle [1,2]. The antiviral activity of phytochemicals is attributed to the different mechanisms that plants utilise when fending off plant viral attacks [3]. The search for better drugs against HIV/AIDS is on-going for many reasons, including the side-effects of current HIV drugs [4] which contributes to patient non-compliance [5] and the rapid development of drug resistant viral strains [6].

Various classes of phytochemicals with potential antiviral activity have been isolated from several hundred plant and herb species [7]. Types of plant constituents isolated include flavonoids, terpenoids, lignans, sulphides, polyphenolics, coumarins, saponins, proteins and chlorophyll derivatives [7,8]. The mechanisms of action of some of these phytochemicals include inhibiting the formation of viral DNA or RNA or inhibiting other viral reproduction steps [7]. Chlorophyll derivatives such as pheophytin-a have been implicated in the inhibition of viral protease from hepatitis C virus [8] while another chlorophyll derivative pheophorbide-a reportedly demonstrated activity against HIV [9], herpes simplex and influenza virus [10].

The *Ocimum* genus is native to many countries and comprises more than 150 species [11]. *Ocimum* species are widely used for the treatment of various ailments and most of these plants are incorporated into indigenous *ayurveda* medicine [12,13]. Plants from the *Ocimum* genus are traditionally used in managing HIV/AIDS and to treat associated opportunistic infections [14,15,16]. *Ocimum gratissimum* [15] *O. suave* [14] and *O. sanctum* [16] are documented as herbal remedies traditionally used for HIV/AIDS management. Leaves are the most commonly used parts of *Ocimum* plants when preparing decoctions for oral administration [14,15,16]. The inhibitory effect of *O. gratissimum* on an HIV-1 strain has been reported [17], while the antibacterial activity of *O. suave* is also documented [18]. Compared to other *Ocimum* plants, a lot more anti-HIV studies have been conducted on *O. sanctum* whereby inhibition of an HIV-1 viral strain [19] and HIV reverse transcriptase [20] was reported.

Due to the various ethnobotanical uses of the *Ocimum* genera, most species of this genus have been extensively studied in vitro, except for *O. labiatum* for which minimal literature exists. *Ocimum labiatum* is found in several southern African countries and this plant has not yet been investigated for anti-HIV-1 activities. Hussein et al. [21] isolated a number of compounds from *O. labiatum* of which 2α-hydroxylabda-8(17),12*E*,14-trien-18-oic acid and labda-8(17),12*E*,14-triene-2*R*,18-diol demonstrated activity against *Mycobacterium tuberculosis* and a human breast cancer cell line, respectively. In a previous study, we reported the antioxidant and anti-inflammatory properties of *O. labiatum* extract and labda-8(17),12*E*,14-triene-2*R*,18-diol [22]. The wide variety of diverse phytochemicals with multi-faceted modes of action that exist in a single plant makes it possible to use extracts and/or isolated compounds to explore various antiviral activities [7]. Here, *O. labiatum* was investigated for inhibitory properties against HIV-1 protease (PR) and reverse transcriptase (RT) with subsequent isolation of the HIV-1 PR inhibiting component. Furthermore, *O. labiatum* extract and isolated compound were evaluated for effects on HIV-1 expression in a chronically infected cell line, and the cytotoxicity of the isolated anti-HIV compound was determined. This study is a first report on anti-HIV properties of *O. labiatum* and the isolated chlorophyll derivative, pheophytin-a.

## 2. Results

### 2.1. Structural Elucidation of Pheophytin-a

**Figure 1:**
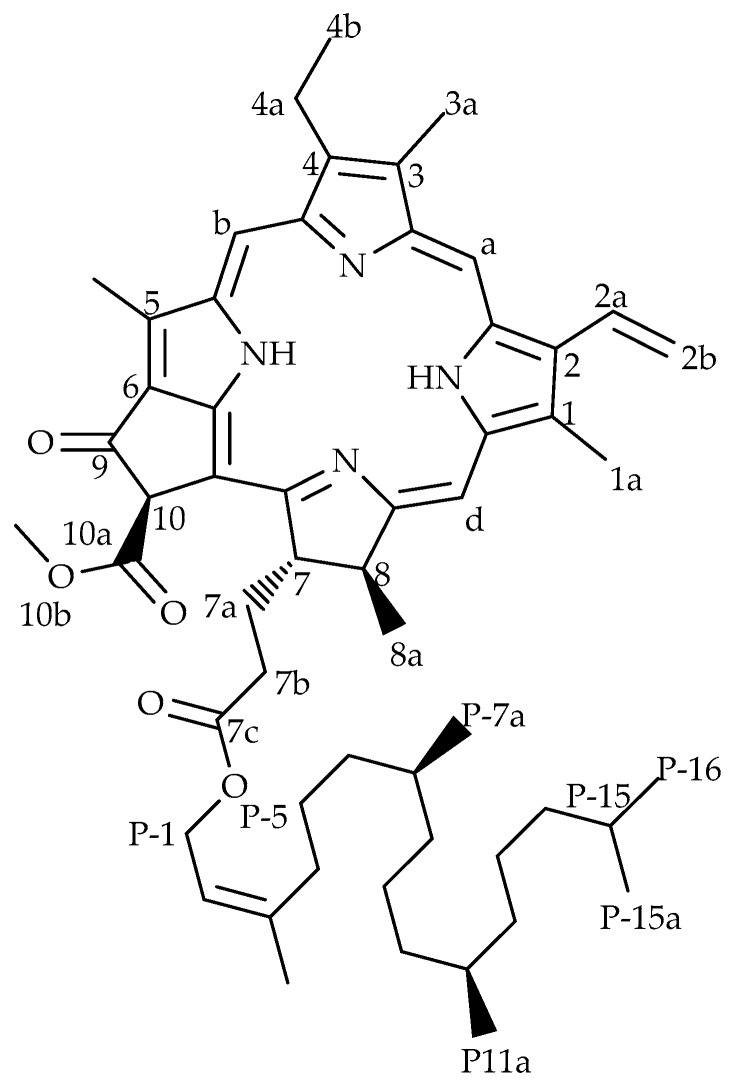
Chemical structure of a chlorophyll derivative pheophytin-a.

The proton NMR spectrum of the isolated compound was similar to the spectrum of pheophytin-a (phy-a) (Figure 1). The chemical shifts (Table 1) were in agreement with those reported in the literature [8,23] and the propionic side chains in the spectrum (2.0–2.7 ppm) were identical to those illustrated by Smith et al. [23]. The proton resonances were properly assigned, except for protons of the phytyl ester (side-chain) and this is also in accordance with what Smith and co-workers [23] found when they could not assign with confidence all protons of the phytyl ester. A thorough literature search indicated similar data; most studies reporting phy-a either did not take into account the protons of the chain and the reported molecular weights varied [8,24] and this explains the complexity of this compound especially in its phytyl side-chain. Based on the exact and isotopic masses of the compound, the molecular formula was suggested to be C_55_H_74_N_4_O_5_ (MW = 871.20). NMR spectra for phy-a are provided as supplementary data.

### 2.2. HIV-1 PR Inhibition

**Figure 2:**
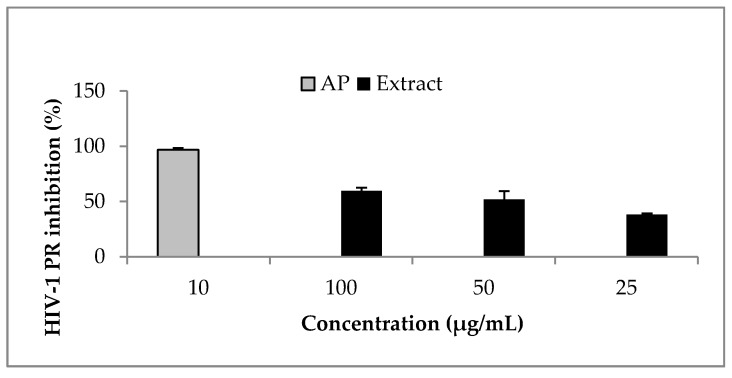
Effect of *O. labiatum* extract on HIV-1 PR. The extract, fluorogenic substrate and HIV-1 PR were incubated at 37 °C for 1 h. The extract significantly (*p* < 0.05) inhibited HIV-1 PR with >50% inhibition at 50 and 100 μg/mL. Acetyl pepstatin (AP) a known protease inhibitor was used as control (10 μg/mL = 15.5 μM).

**Figure 3:**
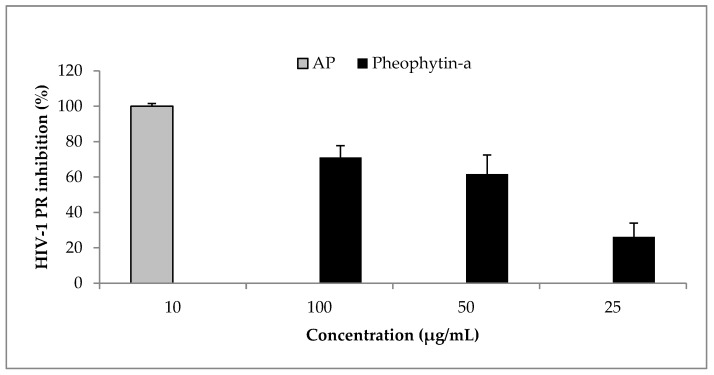
Effect of phy-a on HIV-1 PR. Phy-a, fluorogenic substrate and HIV-1 PR were incubated at 37 °C for 1 h. Phy-a significantly (*p* < 0.05) inhibited HIV-1 PR with >50% inhibition at 50 and 100 μg/mL. The estimated IC_50_ value was 44.4 ± 1.5 μg/mL (51 ± 1.7 μM). Acetyl pepstatin (AP) a known protease inhibitor was used as control 10 μg/mL = 15.5 μM.

*Ocimum labiatum* extract inhibited HIV-1 PR by more than 50% at both 50 and 100 μg/mL (Figure 2) and the estimated IC_50_ value of the crude extract was 49.8 ± 0.4 μg/mL. Weak inhibitory activity of the extract was obtained against HIV-1 RT when the highest concentration tested for the extract (100 μg/mL) inhibited by 21% and this led to the HIV-1 PR fluorogenic assay being used as the guiding assay in the purification of the crude extract in order to obtain the specific component responsible for HIV-1 PR inhibition. The isolated compound, phy-a, inhibited HIV-1 PR (Figure 3) with an estimated IC_50_ value of 44.4 ± 1.5 μg/mL (51 ± 1.7 μM).

### 2.3. Inhibition of PMA-Induced HIV-1 Expression by O. labiatum

**Figure 4:**
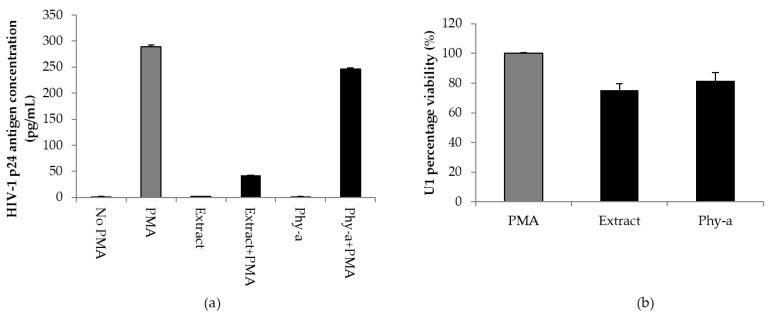
Effect of *O. labiatum* extract and phy-a on HIV-1 replication in U1 cells. (**a**) Cells were pre-treated with 25 μg/mL of extract and 12.5 μg/mL (14.3 μM) of phy-a for 6 h before viral stimulation with PMA. Controls included unstimulated U1 cells (No PMA), PMA stimulated cells (PMA) and unstimulated treated cells (Extract and phy-a only); (**b**) Viability of the tested concentrations in stimulated U1 cells was determined directly after removal of culture supernatant to ensure that inhibition of viral expression was not due to cytotoxicity.

U1 cells are latently infected with HIV-1 and treatment of these cells with PMA activates viral replication [25] which is measurable by assessing the viral core protein, p24, concentration in culture supernatant [26]. The concentration of HIV-1 p24 antigen was significantly (*p* < 0.05) reduced in chronically infected U1 cells in the presence of a non-cytotoxic concentration of the extract (Figure 4a). This potentially means that *O. labiatum* contains compound/s with the ability to inhibit HIV-1 expression. The isolated phy-a also reduced HIV-1 replication, however, the reduction was not significant (*p* > 0.05) meaning that other compounds in *O. labiatum* could have contributed to the reduction of viral expression and not just phy-a. After culture supernatant was collected for HIV-1 p24 testing, the viability of U1 cells that remained after the removal of supernatant was tested by adding MTT. This was done to ensure that the ability of the extract and phy-a to inhibit HIV-1 replication was not due to cytotoxicity (Figure 4b). The extract and phy-a presented moderate cytoxocity; viability of U1 cells was >70%.

### 2.4. Cytotoxic Effect of O. labiatum Extract and Phy-a

Having already demonstrated the limited cytotoxicity of the extract at 25 μg/mL and purified phy-a at 12.5 μg/mL (14.3 μM) in U1 cells (Figure 4), what remained was an assessment of higher concentrations of these plant materials in other types of cells routinely used in HIV drug development studies. In a previous study, we have reported the cytotoxic effect of *O. labiatum* extract on TZM-bl (CC_50_ of 62.6 ± 0.6 μg/mL) and peripheral blood mononuclear cells (PBMCs: CC_50_ of 30.1 ± 0.4 μg/mL) [22]. Here, the CC_50_ value of *O. labiatum* extract in U1 cells was 42.0 ± 0.13 μg/mL which was higher than the concentration of the extract that inhibited HIV-1 replication in U1 cells (25 μg/mL). The CC_50_ value of phy-a in U1 cells, 51.3 ± 1.0 μg/mL (58.9 ± 1.2 μM), was also higher than the concentration tested for potential inhibition of HIV-1 replication (12.5 μg/mL = 14.3 μM) in U1 cells.

**Figure 5:**
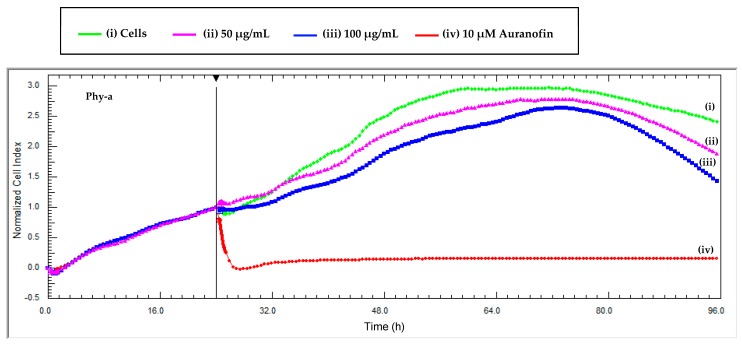
Real time cytotoxicity monitoring of phy-a on TZM-bl cells. The cells were seeded at 10,000 cells per well and were exposed to two concentrations of the compound at 24 h when the cell index was ±1. (i) A control of untreated cells was included and (iv) auranofin, a positive control for cytotoxicity. Each data point was calculated from duplicate values normalized against the time just before sample addition. Phy-a demonstrated low cytotoxicity towards TZM-bl at both concentrations tested, 50 and 100 μg/mL (57 and 115 μM).

The intrinsic colour of phy-a interfered with the absorbance of tetrazolium dyes in the adherent TZM-bl cell line at high concentrations (≥50 μg/mL) and that is why a dye-free technique, RT-CES, was directly used to determine the viability of TZM-bl cells in the presence of phy-a. Phy-a demonstrated low cytotoxicity (Figure 5) at both concentrations tested (50 and 100 μg/mL = 57 and 115 μM). At 96 h, the cell index for control cells (i) was 2.4 and that of phy-a was 1.8 ((ii) 50 μg/mL) and 1.3 ((iii) 100 μg/mL) and this indicated that viability of both concentrations of phy-a was >50% because the cell indexes were more than half the cell index of control cells (2.4/2). The cell index (*y*-axis) is directly correlated to the number of cells in the wells and the higher the cell index the more viable the cells [27].

## 3. Discussion

Even though *O. labiatum* has not been reported to be used traditionally in HIV/AIDS, similar plants *O. sauve*, *O. sanctum* and *O. gratissimum* are administered traditionally to HIV/AIDS patients to manage the disease and related illnesses [14,15,16]. The antiviral and antimicrobial properties reported on for plants from the *Ocimum* genus could mean that these species generally contain antiviral properties as proven by the data presented here for one of those species. In vitro anti-HIV activity of *O. sanctum* and *O. gratissimum* has been reported. Various *O. sanctum* extracts reportedly inhibited HIV-1 RT with the lowest IC_50_ value of 72.22 ± 6.04 μg/mL [19,20], the ability of *O. samctum* extract to inhibit an HIV strain was also reported by Rege et al. [19]. *O. gratissimum* extract reportedly inhibited an HIV strain in vitro [17].

Other studies have also reported on the anti-HIV potential of Lamiaceae family plants, extracts and compounds, observed through in vitro inhibition of HIV strains or enzymes [28,29,30,31]. In a previous study, we have shown the anti-HIV activity of a Lamiaceae plant, *Plectranthus barbatus*, which is popularly used in traditional settings for HIV/AIDS management [29].

Presented here is the first report of the ability of another member of the Lamiaceae family, *O. labiatum* extract, to inhibit HIV-1 expression in vitro which supports emerging evidence of the ability of Lamiaceae plants to reduce HIV-1 replication either by hindering the whole virus or through direct inhibition of HIV-1 enzymes [28,29,30]. The crude extract of *O. labiatum* and isolated phy-a inhibited HIV-1 PR, and also suppressed viral replication in a chronically infected HIV-cell line model.

In vitro cytotoxicity of *O. labiatum* extract and other compounds isolated from the plant was previously reported [21,22]. Cytotoxicity varies in compounds isolated from *O. labiatum*. The chlorophyll derivative, phy-a, isolated from *O. labiatum* in this study was generally non-cytotoxic at high concentrations. The non-cytotoxic effect of phy-a is in agreement with a report by Fang et al. [32], whereby phy-a isolated from a brown alga was inactive against selected cancer cell lines. A labdane diterpenoid compound previously isolated from *O. labiatum* reportedly presented moderate cytotoxicity against breast cancer cells (MCF-7), TZM-bl and PBMCs [21,22], whereas another diterpenoid from the same plant did not present any cytotoxicity at the highest concentration tested in MCF-7 [21]. Oleanolic acid which is commonly isolated from *Ocimum* plants, including *O. labiatum*, presented strong toxicity against solid tumor cancer cell lines [13,21]. Cytotoxicity of *O. labiatum* extract at high concentrations could be attributed to oleanolic acid.

Chlorophyll is the most widely distributed natural pigment with strong antioxidant activity found in leaves and most other plant parts [33]. Chlorophyll is highly unstable and its breakdown leads to the formation of chlorophyll derivatives with the most common derivatives being chlorophyll-a and -b, phy-a and -b as well as pheophorbide-a and -b [33]. The antioxidant activity of phy-a was previously reported [34].

Phy-a isolated from *Lonicera hypoglauca* (Caprifoliaceae), was reported by Wang et al. [8] to be a potent agent against hepatitis C virus (HCV) when it bound to the active site of HCV-NS3 protease. Makatini et al. [35] worked with compounds structurally similar to phy-a and explained the mechanism of action against HIV PR of long chain inhibitors with carbonyl groups. According to the findings of Makatini and colleagues [35], HIV-1 PR converts ketones of long chain inhibitors to hydroxyl groups, and the newly formed hydroxyl group binds to the active pocket of the enzyme [35]. Phy-a contains regions with ketones which possibly inhibited HIV-1 PR in this study. In another study, three pheophytin compounds isolated from *Clinacanthus lutans* (Acanthaceae) reportedly demonstrated anti-herpes simplex viral (HSV) activity; the three pheophytins inactivated HSV before cell entry [36]. Pheophorbide-a (pheo-a), is another chlorophyll derivative with reported anti-HIV activity. According to Zhang et al. [9], pheo-a which is relatively abundant in green plants was purified from *Vatica cinerea* (Dipterocarpaceae) and was able to inhibit HIV infectivity in HOG.R5 cells. Pheo-a isolated from another natural source, *Opuntia ficus-indica* (Cactaceae), reportedly demonstrated potent virucidal effects on HSV and influenza A virus [10]. The results of the current and previous studies all point towards the potential virucidal effects of chlorophyll derivatives against enveloped viruses; HIV, HSV and HCV.

## 4. Materials and Methods

### 4.1. General Experimental Procedures

^1^H-NMR spectra were recorded on a Bruker 400 MHz spectrometer (Bruker, Billerica, MA, USA), using tetramethylsilane as internal standard and deuterated chloroform for dissolution of the sample. Thin layer chromatography (TLC) was carried out on Merck (Darmstadt, Germany) silica gel plates F_254_ (0.25 mm layer thickness) and visualized using UV lamp at 254 and 360 nm, and by spraying with vanillin-sulphuric acid. Column chromatography separations and purifications were performed on silica gel 60 (70–230 mesh) from Merck and on Sephadex LH-20 (Sigma, St. Louis, MO, USA). Prep-HPLC analysis was conducted to further purify the bioactive fraction. This was done with a 6AD preparative LC system equipped with a UV-visible (254 and 370 nm) detector, a 10AF manual injector, and a FRC-10A fraction collector (Shimadzu, Kyoto, Japan). Aliquots (200 μL) were injected in a C18 Jupiter analytical column of 250 mm × 4.6 mm × 10 μm (particle size).

### 4.2. Plant Material

Fresh aerial parts of *O. labiatum* were collected during February 2012 from the Botanical Garden of the University of Pretoria (S25°45′21′′ E28°13′51′′). The specimen was authenticated at the H.G.W.J. Schweikerdt Herbarium of the University of Pretoria where the voucher specimen (117693) is deposited. The leaves were separated from the stems and extracted while fresh.

### 4.3. Extraction and Compound Isolation

Fresh leaves (894.6 g) were extracted with ethanol (10 L) at room temperature and filtered. The filtrate was concentrated in vacuo at 50 °C. The concentrated residue was dissolved in ethyl acetate (EtOAc) in order to obtain a fraction that excludes polar tannins regarded as non-specific enzyme inhibitors [29,30]. The EtOAc fraction was evaporated to dryness under reduced pressure, yielding a residue of 20.7 g. The residue was then subjected to column chromatography (silica gel 70–230 mesh) and eluted with a mixture of hexane-EtOAc of increasing polarity. The HIV-1 enzyme inhibiting fraction was eluted with 30–50% EtOAc. This fraction was further subjected to gel filtration chromatography (Sephadex LH-20) eluting with chloroform from which a sub-fraction (981.9 mg) was obtained. The sub-fraction was further purified with prep-HPLC by eluting sample isocratically with acetonitrile/methanol (1:1, *v*/*v*) at a flow rate of 1.0 mL/min for 30 min to afford a compound (10 mg) with spectral data in agreement with those of pheophytin-a [23].

### 4.4. HIV-1 PR Assay

The method followed to test *O. labiatum* extract and the isolated phy-a against HIV-1 PR was previously described [29,37]. This procedure uses a fluorogenic HIV Protease Substrate 1 Arg-Glu(EDANS)-Ser-Gln-Asn-Tyr-Pro-Ile-Val-Gln-Lys(DABCYL)-Arg (Sigma), a synthetic peptide sequence that contains the cleavage site (Tyr-Pro) for HIV protease as well as two covalently modified amino acids for the detection of cleavage. The substrate was dissolved in dimethyl sulfoxide to make a 1 mM stock which was further diluted in PR assay buffer to yield a working solution of 10 μM. The substrate (49 μL) and 2 μL of HIV-1 PR (1 μg/mL; Bachem, Bubendorf, Switzerland) were incubated with samples (25, 50 and 100 μg/mL) for 1 h in black 96 well assay plates. Acetyl pepstatin (AP) was used as a positive control for HIV-1 PR inhibition. Other control wells included substrate only in assay buffer and an untreated enzyme control. The fluorescence intensity was measured in a synergy microplate spectrofluorometer (Thermo Labsystems, Beverly, MA, USA) at an excitation wavelength of 355 nm and an emission wavelength of 460 nm. The 50% inhibitory concentration (IC_50_) was calculated using Graphpad Prism (Graphpad Software Inc., La Jolla, CA, USA). The experiment was performed four times (*n* = 4) in triplicates.

### 4.5. HIV-1 RT Assay

An HIV RT colorimetric assay kit (Roche Diagnostics, Mannheim, Germany) and HIV-1 recombinant RT (Merck) were used to test for the effect of *O. labiatum* extract on HIV-1 reverse transcription. The assay was performed according to the manufacturer’s instructions. The enzyme was incubated for 1 h with three different concentrations of the extract (25, 50 and 100 μg/mL). Subsequent 1 h incubation steps included the binding of biotin-labelled DNA to the surface of microplate modules that have been pre-coated with streptavidin, and the addition of an antibody conjugated to peroxidase that binds to the digoxigenin-labeled DNA. In the final step, the peroxidase substrate solution (2,2′-azino-*bis*-(3-ethylbenzthiazoline-6-sulfonic acid)) was added and the peroxidase enzyme catalyzed the cleavage of the substrate, producing a colored reaction product. The absorbance of the samples was read at 405 nm with a reference wavelength of 492 nm using a microtiter plate reader (Multiskan Ascent; Thermo Labsystems) and was directly correlated to the level of RT activity in the sample. Doxorubicin (Sigma) [29], a known HIV-1 RT inhibitor, was used as a positive control. The experiment was performed for at least four independent biological repeats in triplicates.

### 4.6. HIV-1 p24 Antigen ELISA

The effect of *O. labiatum* extract and the isolated compound, phy-a, on phorbol 12-myristate 13-acetate (PMA; Sigma)-mediated induction of HIV-1 expression was assayed as previously described [25]. U1 cells (latently infected monocytes) were seeded at a concentration of 1 × 10^5^ cells/well and pre-treated with non-cytotoxic concentrations of extract (25 μg/mL) and compound (12.5 μg/mL = 14.3 μM). Previous studies have reported pre-treatment at various times depending on the kind of treatment desired [25,38,39]. Here, pre-treatment was done for 6 h [25] at 37 °C in 90% humidified air with 5% CO_2_. After the 6 h incubation, PMA (2 ng/mL) was added and the samples were further incubated for 66 h. Assay control included PMA-stimulated and unstimulated U1 cells. To monitor HIV-1 activity, the level of HIV-1 p24 antigen was measured in the supernatant using the RETRO-TEK HIV-1 p24 antigen ELISA 2.0 (ZeptoMetrix Corporation, Buffalo, NY, USA). A standard curve was generated using a heat-inactivated HIV-1 p24 antigen standard provided by the manufacturer. The unknown concentration of HIV-1 p24 antigen in the supernatant of extract and compound treated cells was determined by linear regression analysis from the standard curve. The experiment was performed three times (*n* = 3) in triplicates.

### 4.7. Cells

The promonocytic U1 cell line was obtained from the AIDS Research and Reference Reagent Program, NIAID, National Institute of Health (Rockville, MD, USA). U1 cells were maintained at 1 × 10^5^ cells/mL in RPM1 1640 containing 2 mM glutamine (Sigma Aldrich), supplemented with 10% fetal bovine serum (Thermo Scientific, HyClone^®^, Logan, UT, USA), 100 U/mL penicillin and 100 μg/mL streptomycin (Thermo Scientific, HyClone^®^) at 37 °C, 95% humidity and 5% CO_2_. The cells were subcultured every two days. The TZM-bl cell line was also maintained at the same condition at 1 × 10^5^ cells/mL in complete DMEM medium (containing antibiotics and 10% foetal calf serum).

#### Cytotoxicity Analysis of the Extract and Isolated Compound

Cytotoxicity of the samples was assessed using TZM-bl and U1 cells. The cells (1 × 10^4^ cells/well) in complete medium, containing antibiotics and fetal calf serum, were treated with various concentrations of the samples (3.125–100 μg/mL) in 96-well tissue culture plates. Auranofin (10 μM), a positive control for toxicity [40] and untreated cells, were included as experimental controls. Incubation was done for 72 h at 37 °C in a humidified incubator with 5% CO_2_.

The viability of U1 cells was determined by quantifying formazan crystals that form as a result of reduction of 3-(4,5-dimethylthiazol-2-yl)-2,5-diphenyltetrazolium bromide (MTT; Sigma, St. Louis, MO, USA) by dehydrogenases in viable cells. The plates were read at 550 nm (reference wavelength of 690 nm) using a microtiter plate reader (Multiskan Ascent; Thermo Labsystems). The percentage viability was calculated relative to untreated control cells. The 50% cytotoxic concentration (CC_50_) was calculated using Graphpad Prism.

Real-time cell electronic sensing (RT-CES), xCELLigence (Roche Diagnostics) was used to determine the viability of adherent TZM-bl cells in the presence of the bioactive compound. RT-CES only works with adherent cells due to its principle of cell impedance created as cells attach to the surface of interdigitated gold micro-electrodes, integrated on the bottom of special tissue culture plates. The more cells attach to the electrodes, the larger the increases in electrode impedance and the values are displayed as Cell Index (CI) [27]. A detailed procedure was followed as previously described [29]; pre-titrated TZM-bl cells were plated at a concentration of 1 × 10^4^ cells/well and treated with the compound when the cell index was ±1 as per manufacturer’s instructions. Two concentrations of the compound (50 and 100 μg/mL) were tested for their effects on TZM-bl cells in real-time and this was monitored for 72 h. Control wells included 10 μM auranofin and cells in media only. The experiment was performed six times (*n* = 6) in triplicates.

### 4.8. Statistical Analysis

Significant difference, IC_50_ and CC_50_ values were computed using Graphpad Prism 5 and Student’s t test for unpaired observations. A *p* < 0.05 was considered significant.

## 5. Conclusions

Data from the present study provides evidence of phy-a as a potential anti-HIV agent targeting HIV-1 protease. The anti-HIV activity of phy-a supports existing literature reporting on the ability of chlorophyll derivatives to inhibit enveloped viruses including HIV. Identification of phy-a as a potential anti-HIV compound adds to the list of compounds to consider for further development as anti-HIV/AIDS agents, especially in the light of evidence that these types of chlorophyll derivatives inhibit HIV replication.

## Figures and Tables

**Table 1:** Proton NMR data of pheophytin-a.

Position	Isolated ^1^H δ ppm	Literature ^1^H δ ppm [23]
α	9.38	9.38
β	9.52	9.52
δ	8.57	8.55
1a	3.4	3.4
2a	8	8
2b	6.31	6.28
2	6.17	6.18
3a	3.23	3.23
4a	3.69	3.68
4b	1.69	1.69
7	4.21	4.21
7a	2.63	2.63
7a′	2.33	2.34
7b	2.47	2.49
7b′	2.19	2.19
8	4.47	4.46
8a	1.81	1.8
10	6.26	6.26
10b	3.89	3.88
P-5/P-15	1.1, 1.3	1.0–1.3
P-7a, P-11a	0.86	0.85
NH	−1.6	−1.6

## Supplementary Materials

The supplementary materials are available.
