# Supplementary material for: Anti-HIV Activity of Ocimum labiatum Extract and Isolated Pheophytin-a
Source: Molecules. 2017 Nov 6;22(11):1763. doi: 10.3390/molecules22111763 (PMC6150305; doi:10.3390/molecules22111763)
Supplement: Supplementary file 1 [file molecules-22-01763-s001.pdf]

PK3 (Phy-a)

Current Data Parameters	
NAME	PK03
EXPNO	1
PROCNO	1

F2 - Acquisition Parameters	
Date_	20120905
Time	11.08
INSTRUM	spect
PROBHD	5 mm QNP 1H/15
PULPROG	zg30
TD	65536
SOLVENT	MeOD
NS	16
DS	2
SWH	6172.839 Hz
FIDRES	0.094190 Hz
AQ	5.3084660 sec
RG	228.1
DW	81.000 usec
DE	6.00 usec
TE	299.2 K
D1	1.00000000 sec
TD0	1

======== CHANNEL f1 ========	
NUC1	1H
P1	10.00 usec
PL1	0.00 dB
SFO1	300.1318534 MHz

F2 - Processing parameters	
SI	32768
SF	300.1300000 MHz
WDW	EM
SSB	0
LB	0.30 Hz
GB	0
PC	1.00

BRUKER

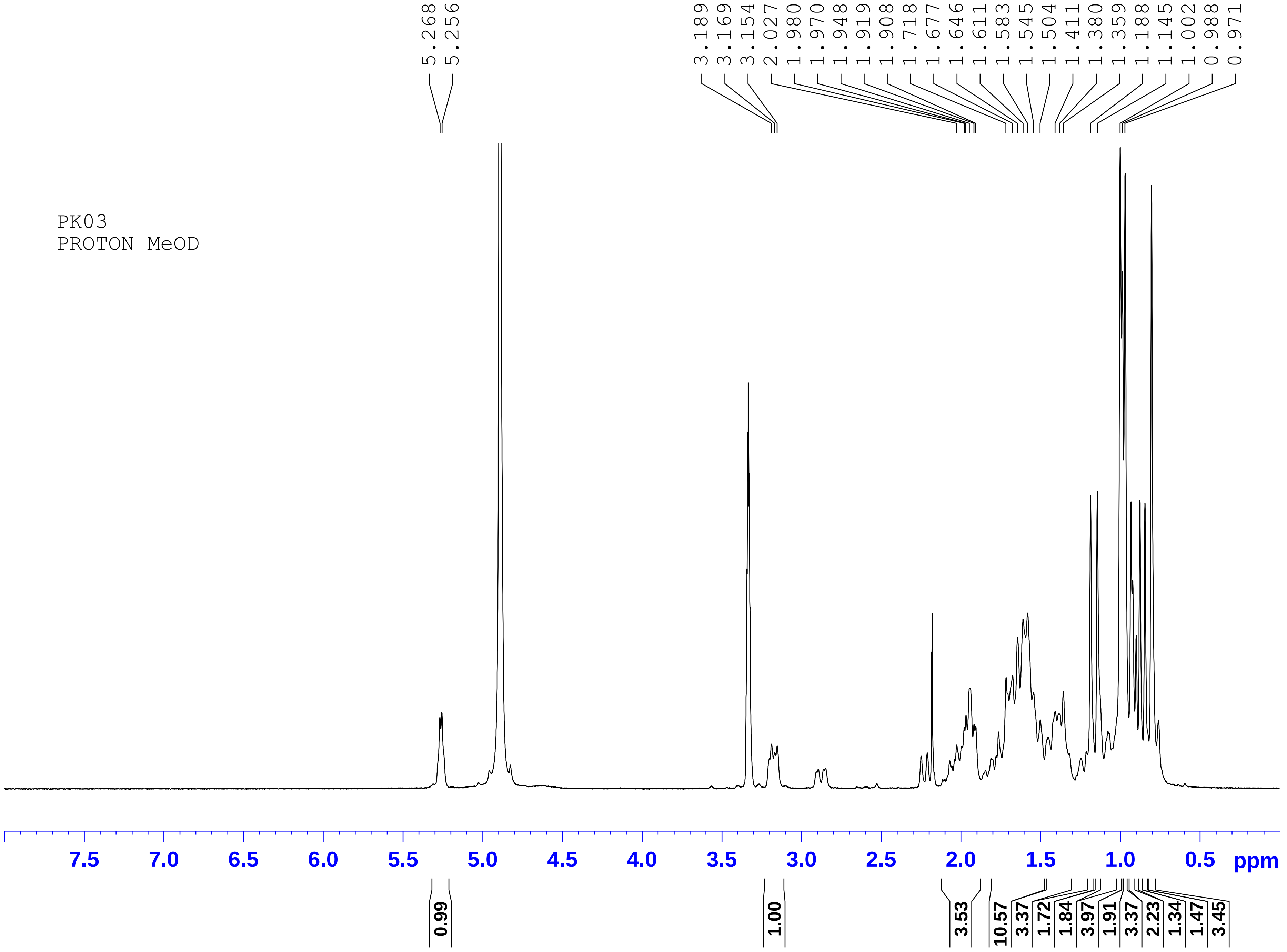
PK03
PROTON MeOD
5.268
5.256
3.189
3.169
3.154
2.027
1.980
1.970
1.948
1.919
1.908
1.718
1.677
1.646
1.611
1.583
1.545
1.504
1.411
1.380
1.359
1.188
1.145
1.002
0.988
0.971
7.5
7.0
6.5
6.0
5.5
5.0
4.5
4.0
3.5
3.0
2.5
2.0
1.5
1.0
0.5
ppm
0.99
1.00
3.53
10.57
3.37
1.72
1.84
3.97
1.91
3.37
2.23
1.34
1.47
3.45

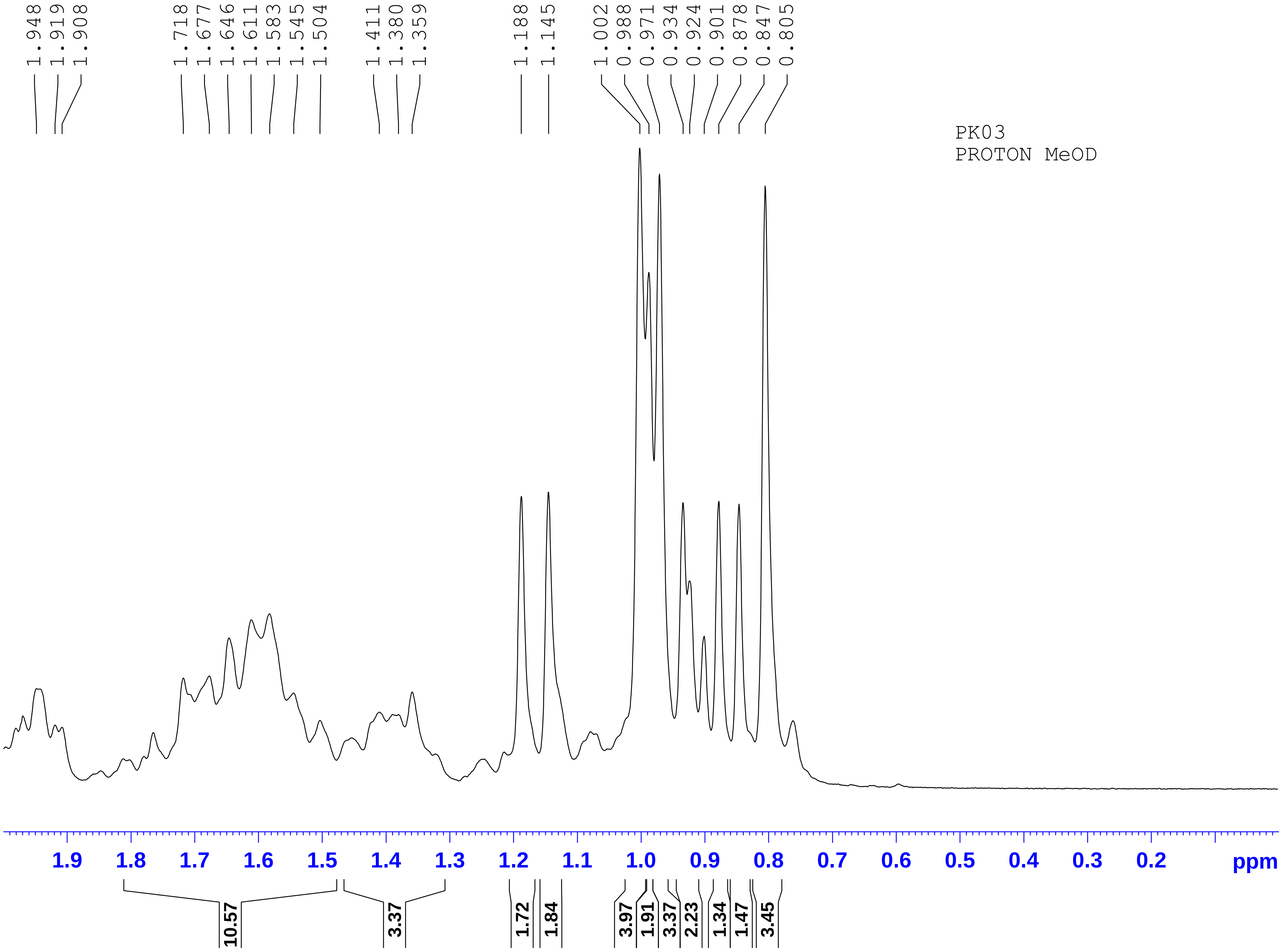

1.948
1.919
1.908
1.718
1.677
1.646
1.611
1.583
1.545
1.504
1.411
1.380
1.359
1.188
1.145
1.002
0.988
0.971
0.934
0.924
0.901
0.878
0.847
0.805
PK03
PROTON MeOD
1.9
1.8
1.7
1.6
1.5
1.4
1.3
1.2
1.1
1.0
0.9
0.8
0.7
0.6
0.5
0.4
0.3
0.2
ppm
10.57
3.37
1.72
1.84
3.97
1.91
3.37
2.23
1.34
1.47
3.45

PK03
C13CPD MeOD

Current Data Parameters

NAME	PK03
EXPNO	2
PROCNO	1

F2 - Acquisition Parameters

Date_	20120907	
Time	3.07	
INSTRUM	spect	
PROBHD	5 mm QNP 1H/15	
PULPROG	zgpg30	
TD	65536	
SOLVENT	MeOD	
NS	4096	
DS	4	
SWH	17985.611	Hz
FIDRES	0.274439	Hz
AQ	1.8219508	sec
RG	14596.5	
DW	27.800	usec
DE	6.00	usec
TE	300.2	K
D1	2.00000000	sec
d11	0.03000000	sec
DELTA	1.89999998	sec
TD0	1	

======== CHANNEL f1 ========

NUC1	13C	
P1	8.50	usec
PL1	-2.00	dB
SFO1	75.4752953	MHz

======== CHANNEL f2 ========

CPDPRG2	waltz16	
NUC2	1H	
PCPD2	80.00	usec
PL2	0.00	dB
PL12	18.06	dB
PL13	18.00	dB
SFO2	300.1312005	MHz

F2 - Processing parameters

SI	32768	
SF	75.4677490	MHz
WDW	EM	
SSB	0	
LB	1.00	Hz
GB	0	
PC	1.40	

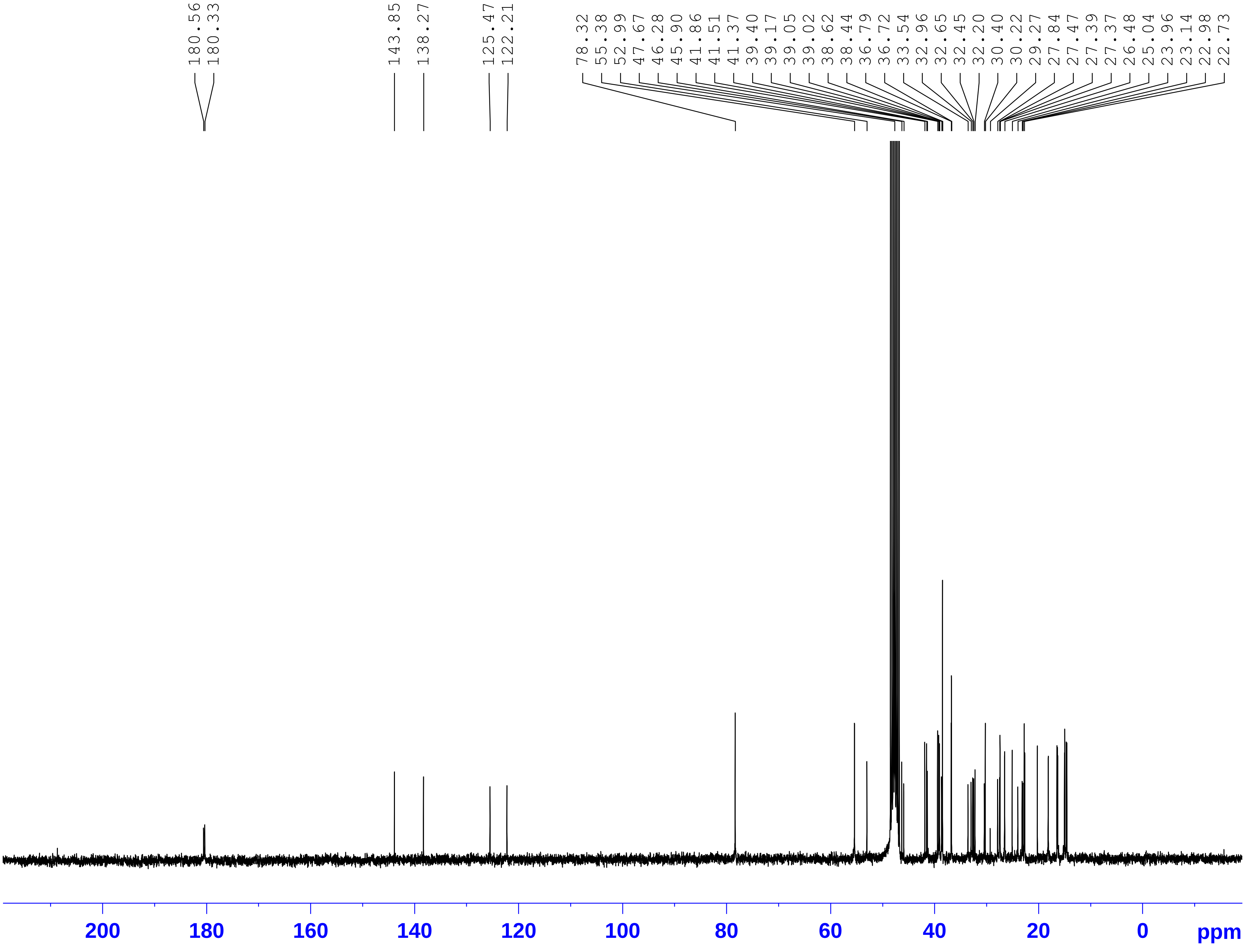
180.56
180.33
143.85
138.27
125.47
122.21
78.32
55.38
52.99
47.67
46.28
45.90
41.86
41.51
41.37
39.40
39.17
39.05
39.02
38.62
38.44
36.79
36.72
33.54
32.96
32.65
32.45
32.20
30.40
30.22
29.27
27.84
27.47
27.39
27.37
26.48
25.04
23.96
23.14
22.98
22.73
200
180
160
140
120
100
80
60
40
20
0
ppm

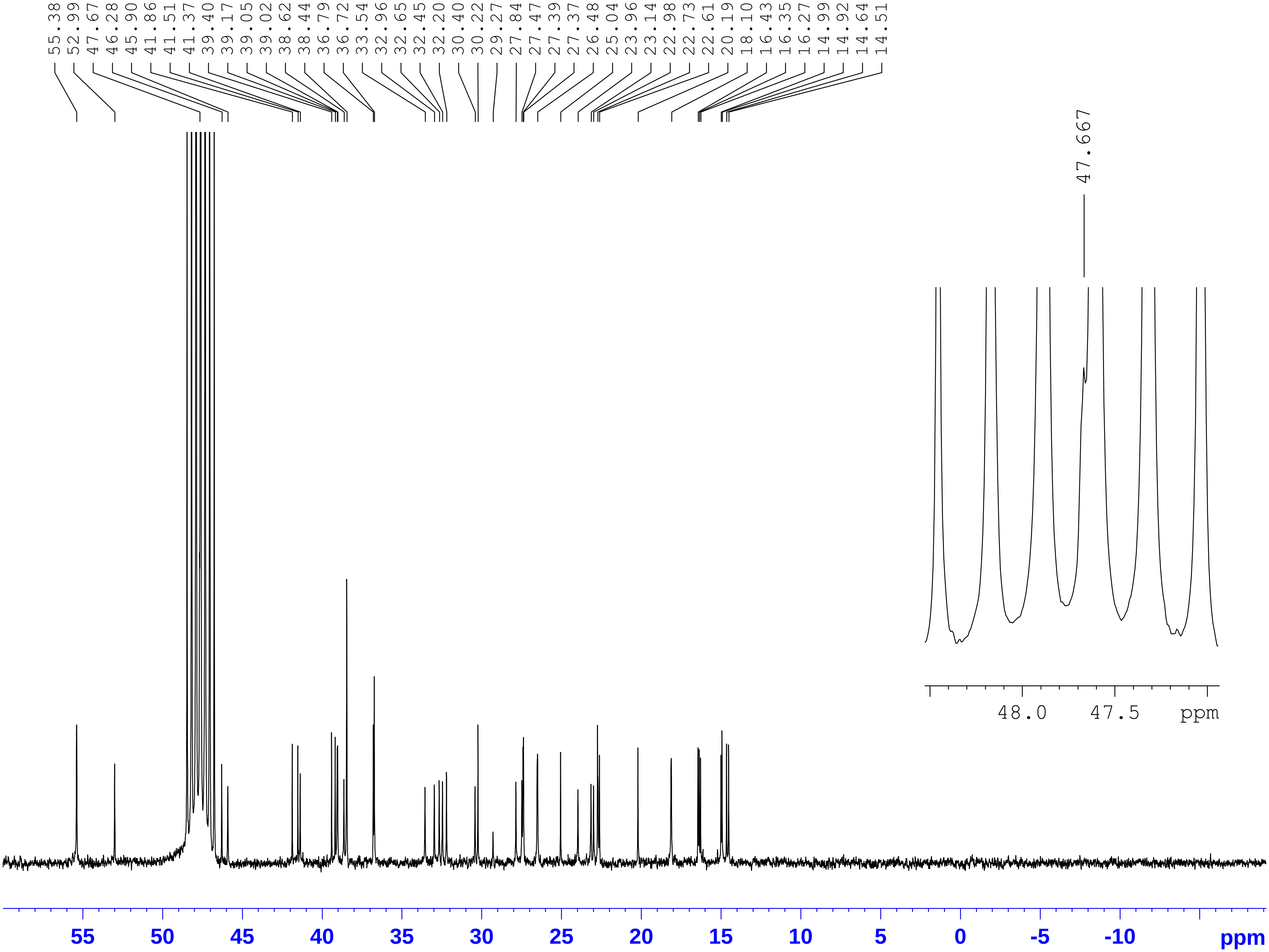
55.38
52.99
47.67
46.28
45.90
41.86
41.51
41.37
39.40
39.17
39.05
39.02
38.62
38.44
36.79
36.72
33.54
32.96
32.65
32.45
32.20
30.40
30.22
29.27
27.84
27.47
27.39
27.37
26.48
25.04
23.96
23.14
22.98
22.73
22.61
20.19
18.10
16.43
16.35
16.27
14.99
14.92
14.64
14.51
47.667
48.0
47.5
ppm
55
50
45
40
35
30
25
20
15
10
5
0
-5
-10
ppm

PK03
C13DEPT135 MeOD

```
Current Data Parameters
NAME               PK03
EXPNO                 3
PROCNO                1

F2 - Acquisition Parameters
Date_          20120907
Time               4.14
INSTRUM           spect
PROBHD   5 mm QNP 1H/15
PULPROG         dept135
TD                65536
SOLVENT            MeOD
NS                 1024
DS                    4
SWH           17985.611 Hz
FIDRES         0.274439 Hz
AQ            1.8219508 sec
RG                16384
DW               27.800 usec
DE                 6.00 usec
TE                299.2 K
CNST2       145.0000000
D1           2.00000000 sec
d2           0.00344828 sec
d12          0.00002000 sec
DELTA        0.00001082 sec
TD0                   1

======== CHANNEL f1 ========
NUC1                13C
P1                 8.50 usec
p2                17.00 usec
PL1               -2.00 dB
SFO1         75.4752953 MHz

======== CHANNEL f2 ========
CPDPRG2         waltz16
NUC2                 1H
P3                10.00 usec
p4                20.00 usec
PCPD2             80.00 usec
PL2                0.00 dB
PL12              18.06 dB
SFO2        300.1312005 MHz

F2 - Processing parameters
SI                32768
SF           75.4677490 MHz
WDW                  EM
SSB                   0
LB                 1.00 Hz
GB                    0
PC                 1.40
```

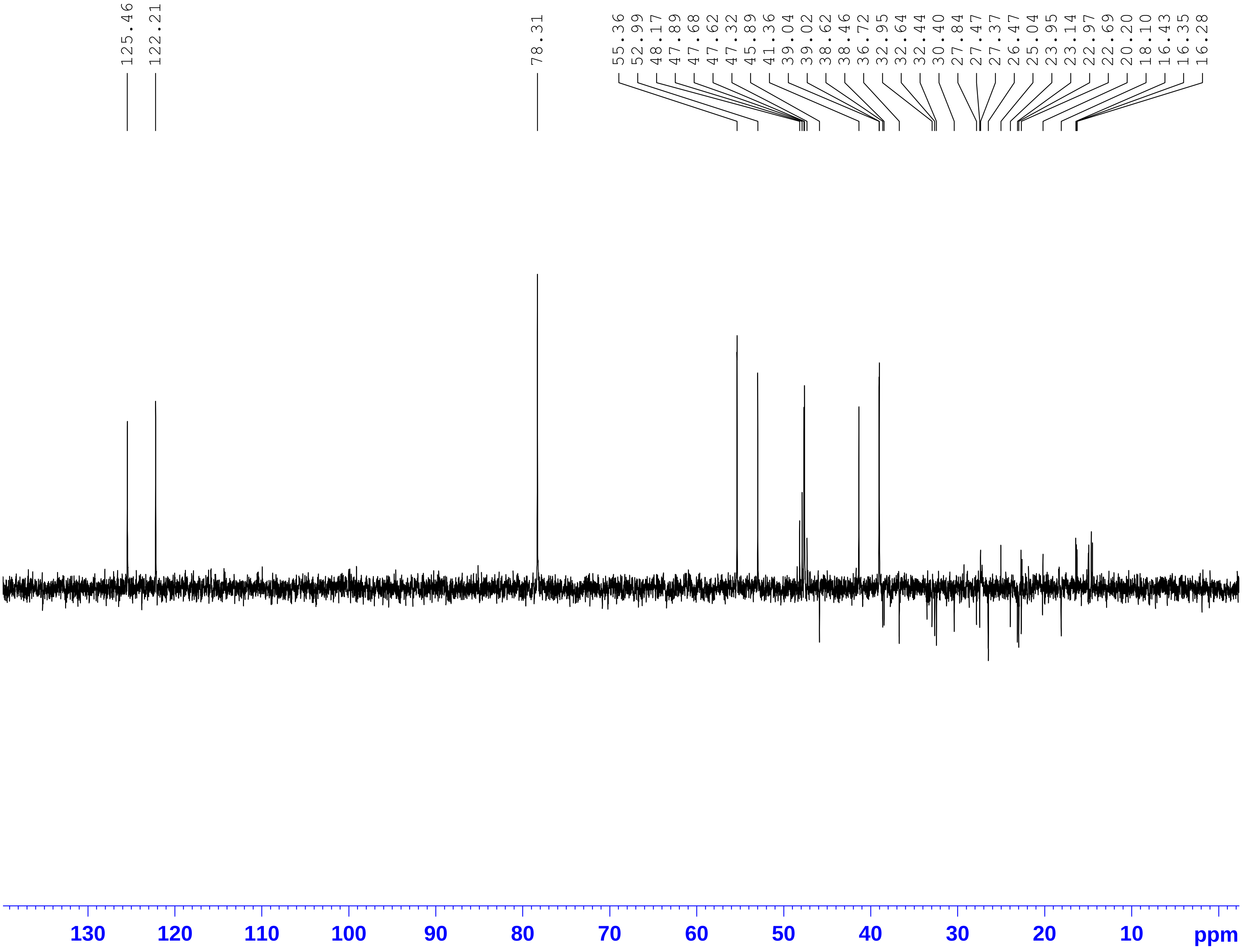
125.46
122.21
78.31
55.36
52.99
48.17
47.89
47.68
47.62
47.32
45.89
41.36
39.04
39.02
38.62
38.46
36.72
32.95
32.64
32.44
30.40
27.84
27.47
27.37
26.47
25.04
23.95
23.14
22.97
22.69
20.20
18.10
16.43
16.35
16.28
130
120
110
100
90
80
70
60
50
40
30
20
10
ppm

PK03
COSYGPSW MeOD

Current Data Parameters

NAME	PK03
EXPNO	4
PROCNO	1

F2 - Acquisition Parameters

Date_	20120907
Time	4.18
INSTRUM	spect
PROBHD	5 mm QNP 1H/15
PULPROG	cosygpqf
TD	2048
SOLVENT	MeOD
NS	6
DS	8
SWH	1698.370 Hz
FIDRES	0.829282 Hz
AQ	0.6029812 sec
RG	64
DW	294.400 usec
DE	6.00 usec
TE	299.2 K
d0	0.00000300 sec
D1	1.14037001 sec
d13	0.00000400 sec
D16	0.00020000 sec
IN0	0.00058880 sec

======== CHANNEL f1 ========

NUC1	1H
P0	10.00 usec
P1	10.00 usec
PL1	0.00 dB
SFO1	300.1308656 MHz

====== GRADIENT CHANNEL =====

GPNAM1	SINE.100
GPNAM2	SINE.100
GPZ1	10.00 %
GPZ2	10.00 %
P16	1000.00 usec

F1 - Acquisition parameters

ND0	1
TD	256
SFO1	300.1309 MHz
FIDRES	6.634256 Hz
SW	5.659 ppm
FnMODE	QF

F2 - Processing parameters

SI	1024
SF	300.1300000 MHz
WDW	SINE
SSB	0
LB	0.00 Hz
GB	0
PC	1.40

F1 - Processing parameters

SI	1024
MC2	QF
SF	300.1300000 MHz
WDW	SINE
SSB	0
LB	0.00 Hz
GB	0

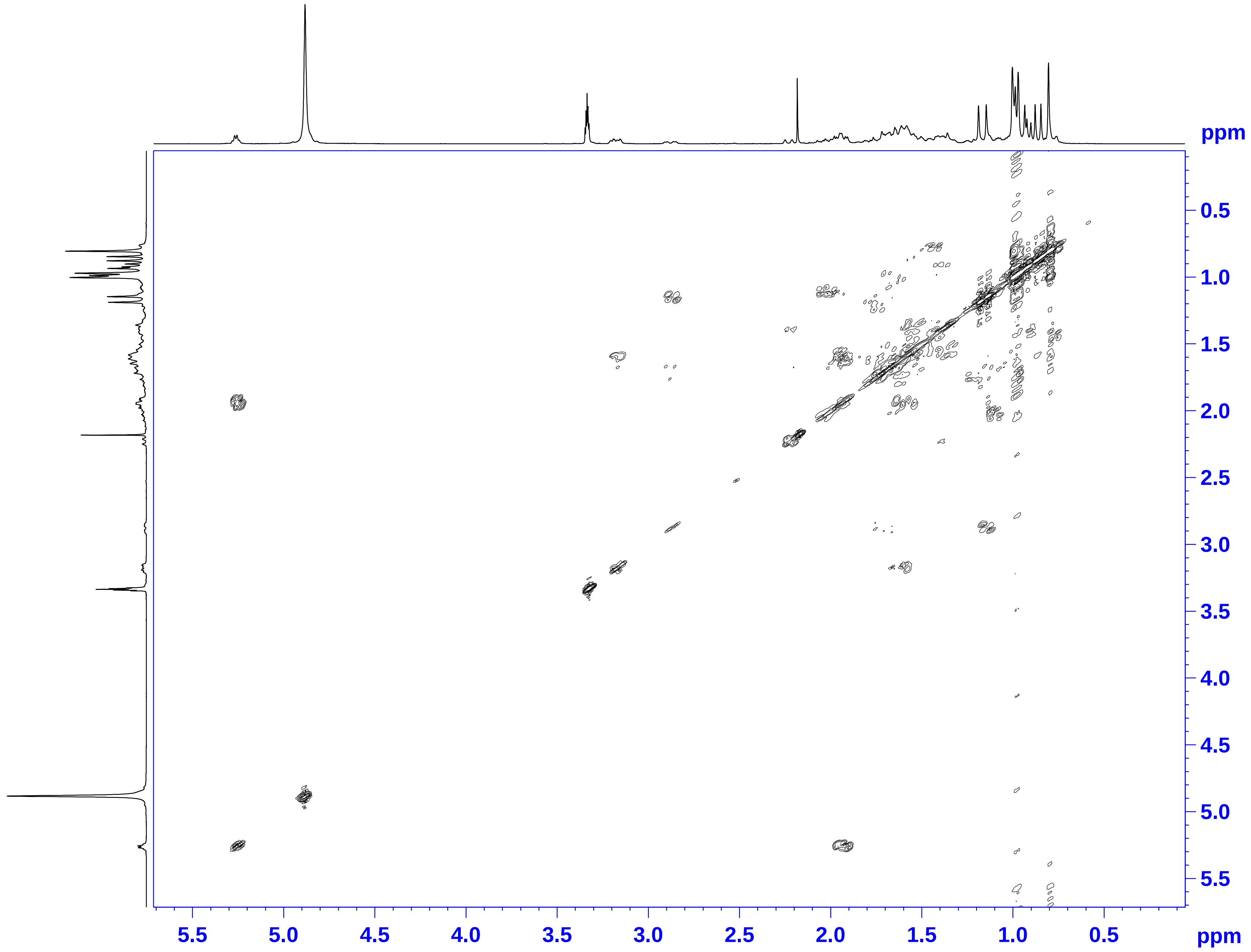

ppm
0.5
1.0
1.5
2.0
2.5
3.0
3.5
4.0
4.5
5.0
5.5
5.5
5.0
4.5
4.0
3.5
3.0
2.5
2.0
1.5
1.0
0.5
ppm

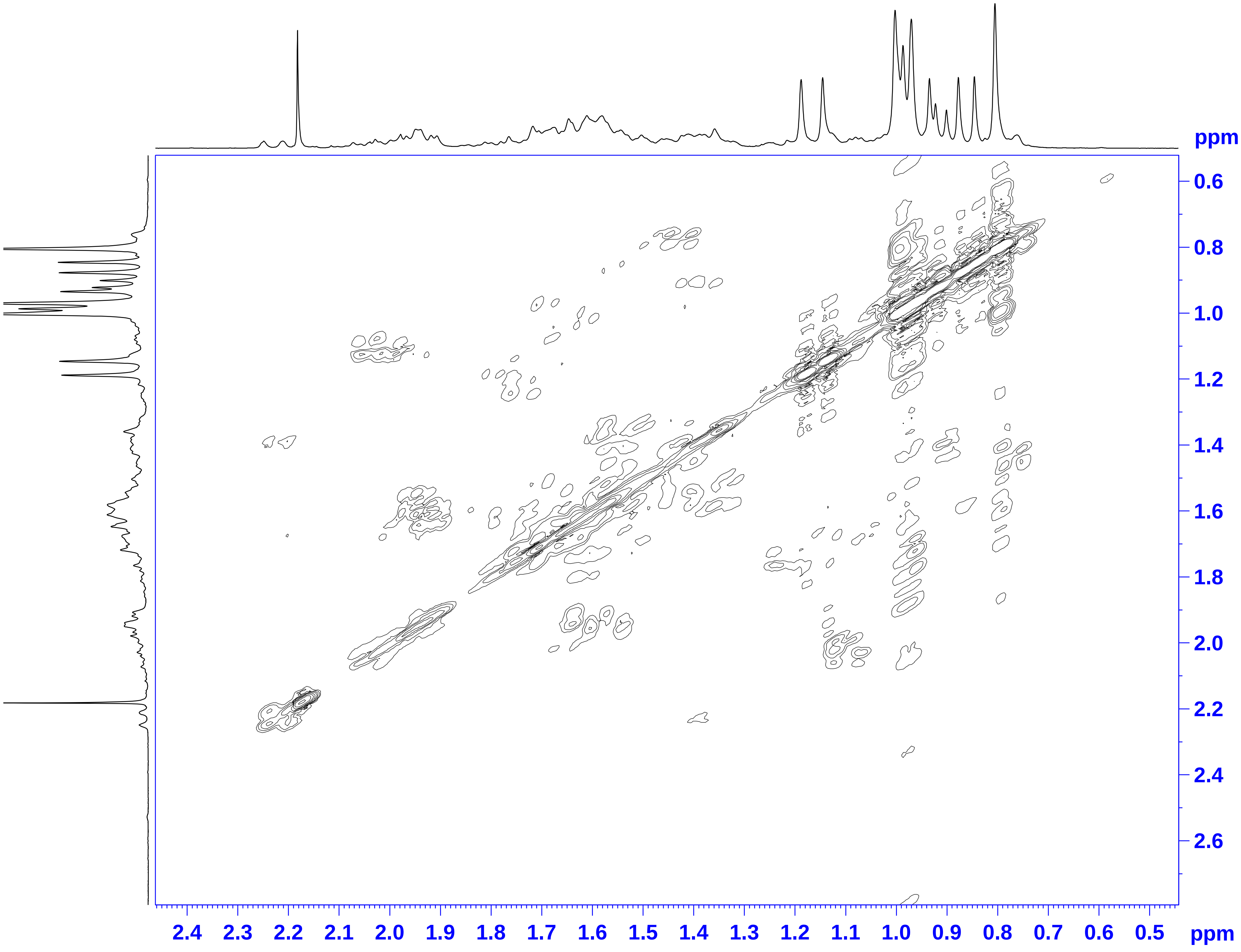

ppm
0.6
0.8
1.0
1.2
1.4
1.6
1.8
2.0
2.2
2.4
2.6
2.4 2.3 2.2 2.1 2.0 1.9 1.8 1.7 1.6 1.5 1.4 1.3 1.2 1.1 1.0 0.9 0.8 0.7 0.6 0.5
ppm

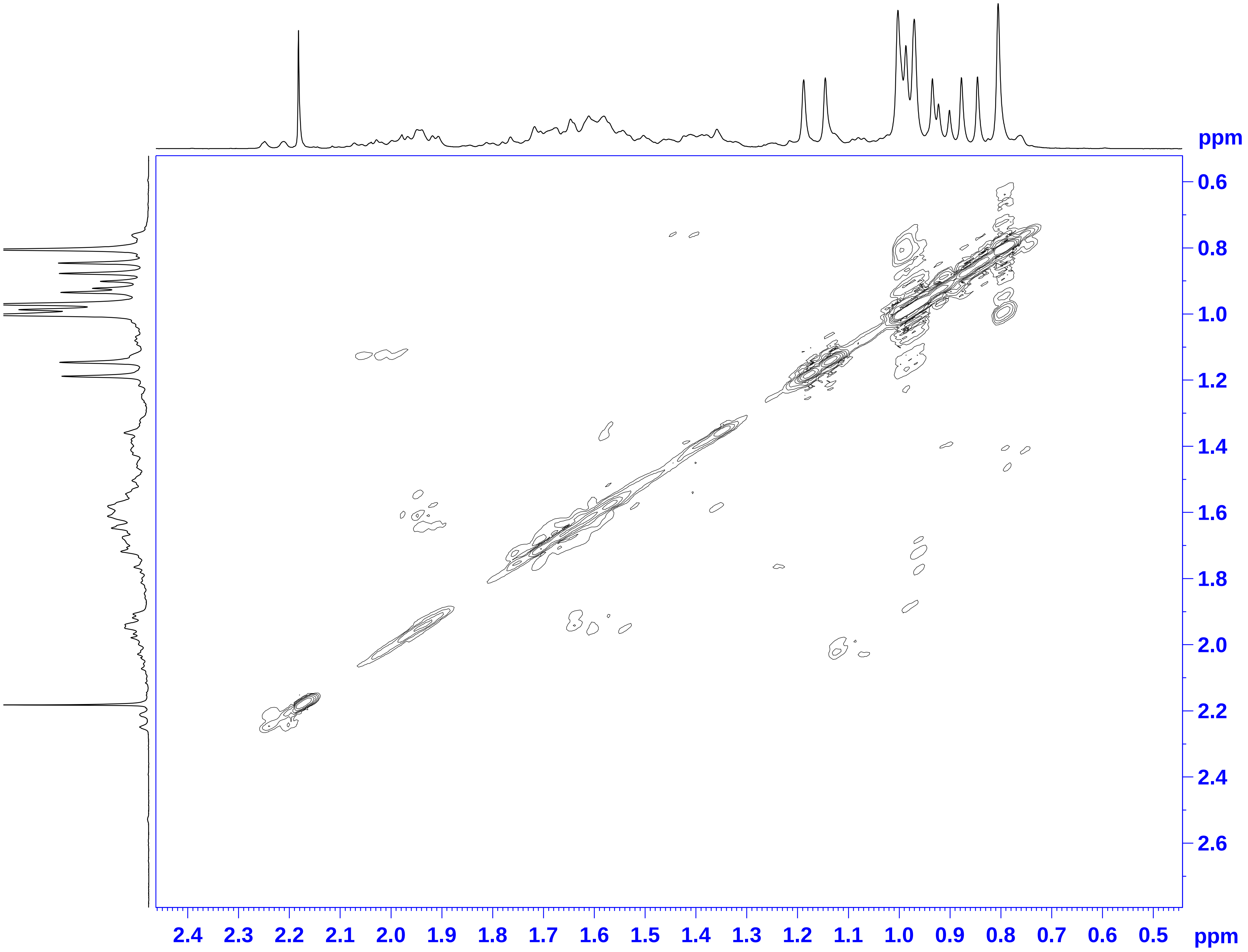
ppm
0.6
0.8
1.0
1.2
1.4
1.6
1.8
2.0
2.2
2.4
2.6
2.4 2.3 2.2 2.1 2.0 1.9 1.8 1.7 1.6 1.5 1.4 1.3 1.2 1.1 1.0 0.9 0.8 0.7 0.6 0.5
ppm

PK03
HMQCGP MeOD

Current Data Parameters

NAME	PK03
EXPNO	5
PROCNO	1

F2 - Acquisition Parameters

Date_	20120907	
Time	5.06	
INSTRUM	spect	
PROBHD	5 mm QNP 1H/15	
PULPROG	hmqcgpqf	
TD	1024	
SOLVENT	MeOD	
NS	24	
DS	16	
SWH	1698.370	Hz
FIDRES	1.658564	Hz
AQ	0.3015156	sec
RG	26008	
DW	294.400	usec
DE	6.00	usec
TE	299.2	K
CNST2	145.0000000	
d0	0.00000300	sec
D1	1.36728895	sec
d2	0.00344828	sec
d12	0.00002000	sec
d13	0.00000400	sec
D16	0.00020000	sec
DELTA1	0.00222428	sec
IN0	0.00004000	sec

======== CHANNEL f1 ========

NUC1	1H	
P1	10.00	usec
p2	20.00	usec
PL1	0.00	dB
SFO1	300.1308656	MHz

======== CHANNEL f2 ========

CPDPRG2	garp	
NUC2	13C	
P3	8.50	usec
PCPD2	80.00	usec
PL2	-2.00	dB
PL12	17.47	dB
SFO2	75.4734083	MHz

====== GRADIENT CHANNEL =====

GPNAM1	SINE.100	
GPNAM2	SINE.100	
GPNAM3	SINE.100	
GPZ1	50.00	%
GPZ2	30.00	%
GPZ3	40.10	%
P16	1000.00	usec

F1 - Acquisition parameters

ND0	2	
TD	206	
SFO1	75.47341	MHz
FIDRES	60.679611	Hz
SW	165.621	ppm
FnMODE	QF	

F2 - Processing parameters

SI	1024	
SF	300.1300000	MHz
WDW	QSINE	
SSB	2	
LB	0.00	Hz
GB	0	
PC	1.40	

F1 - Processing parameters

SI	1024	
MC2	QF	
SF	75.4677490	MHz
WDW	QSINE	
SSB	2	
LB	0.00	Hz
GB	0	

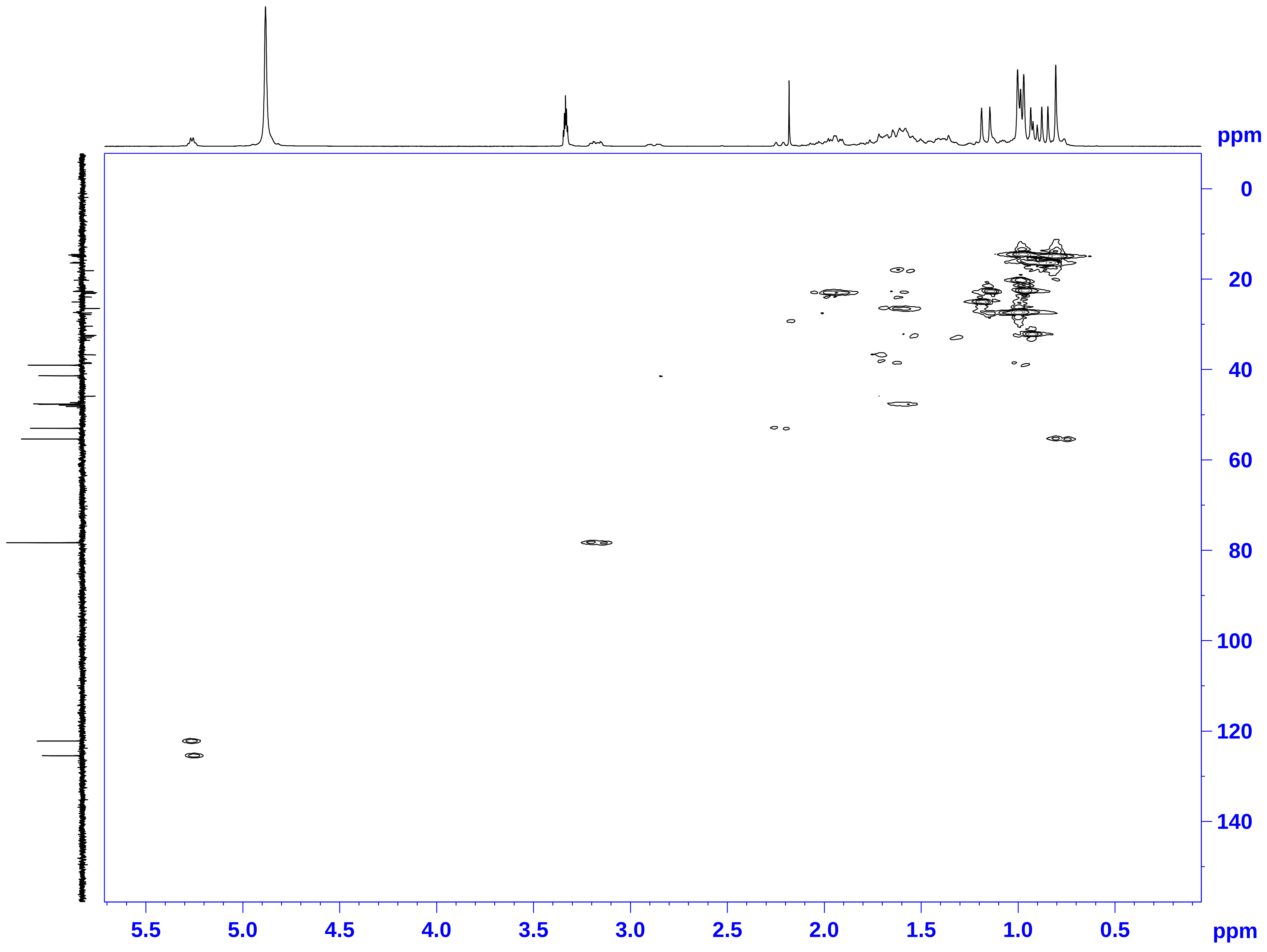
ppm
0
20
40
60
80
100
120
140
5.5
5.0
4.5
4.0
3.5
3.0
2.5
2.0
1.5
1.0
0.5
ppm

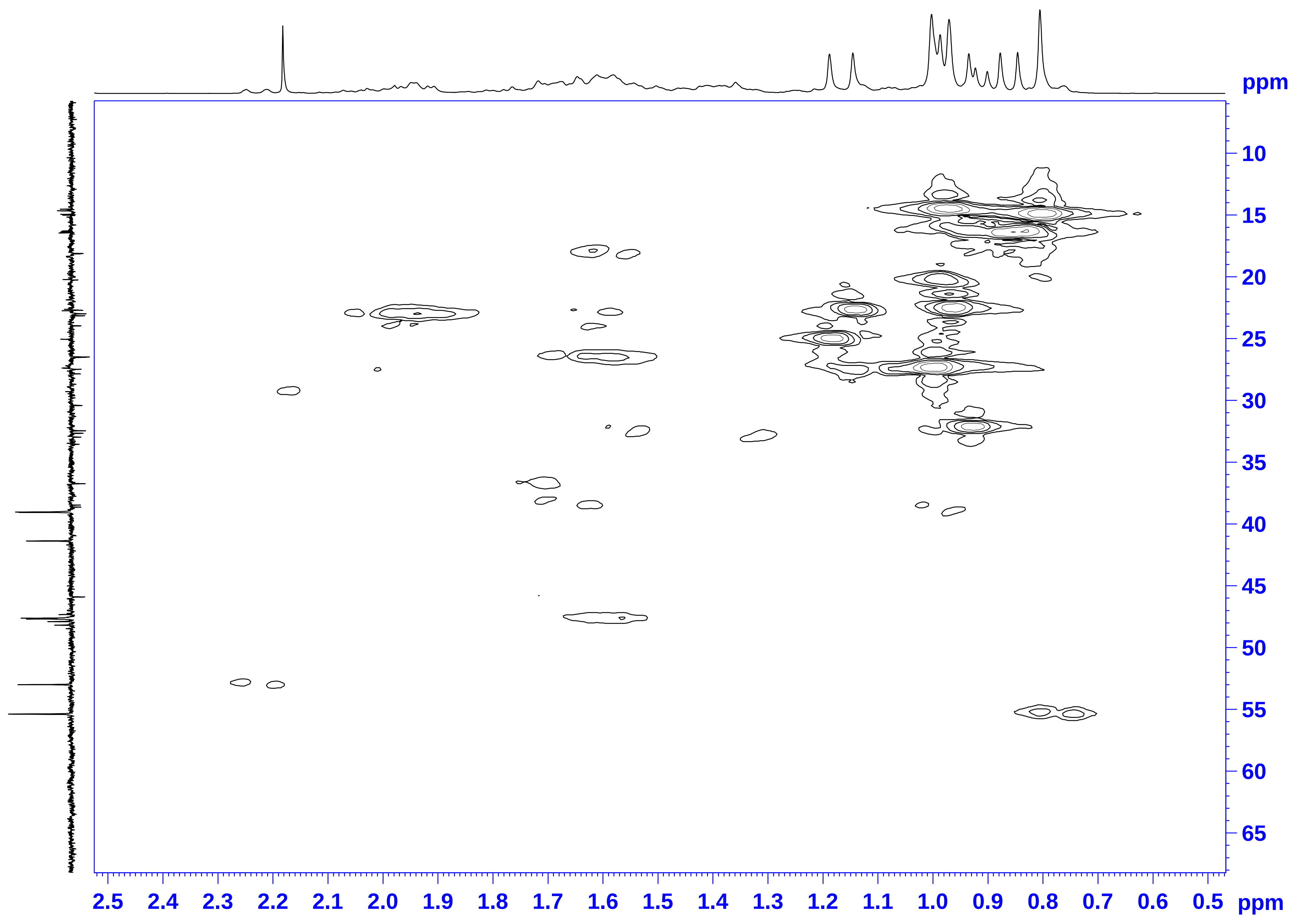
ppm
10
15
20
25
30
35
40
45
50
55
60
65
2.5 2.4 2.3 2.2 2.1 2.0 1.9 1.8 1.7 1.6 1.5 1.4 1.3 1.2 1.1 1.0 0.9 0.8 0.7 0.6 0.5
ppm
